# Serum Brain-derived Neurotrophic Factor Levels as a Biomarker of Treatment Response in Patients With Depression: Systematic Review and Meta-analysis

**DOI:** 10.62641/aep.v53i4.1967

**Published:** 2025-08-05

**Authors:** Yang Li, Jing Ma, Wen-xiu Zhang, Yan-li Cao, Chen Lei

**Affiliations:** ^1^The First Clinical Medical College of Ningxia Medical University, 750004 Yinchuan, Ningxia, China; ^2^Department of Geriatrics and Special Needs, General Hospital of Ningxia Medical University, 750004 Yinchuan, Ningxia, China

**Keywords:** brain-derived neurotrophic factor, depression, antidepressive agents, meta-analysis, biomarkers

## Abstract

**Background:**

Brain-derived neurotrophic factor (BDNF) plays a key role in the pathophysiology of depression and the mechanism of action of antidepressants. This study aimed to evaluate the changes of BDNF in patients with depression and how it is affected by antidepressant treatment through meta-analysis.

**Methods:**

Multiple databases (including PubMed, Embase and China National Knowledge Infrastructure (CNKI)) were searched for studies on BDNF levels in patients with depression published up to November 15, 2024. Meta-analyses of serum and plasma BDNF levels were performed using RevMan 5.4.1, with the effect sizes expressed as mean differences (MD) and 95% confidence intervals. Heterogeneity was assessed using I^2^ statistics (random-effects model if I^2^ ≥ 50%; fixed-effects if I^2^ < 50%).

**Results:**

Serum BDNF levels in patients with depression were significantly lower than those in healthy controls [MD = –1.54, 95% confidence intervals (CI) (–2.85 to –0.24), *p* = 0.02]. Antidepressant drug treatment for 6 weeks significantly increased serum BDNF levels [MD = 7.42, 95% CI (1.10–13.74), *p* = 0.02], but the effect of 4 weeks of treatment was not statistically significant. Plasma BDNF levels showed no statistically significant differences between depressed patients and healthy controls (*p* > 0.05). Sensitivity analysis indicated that the meta-analysis results were robust and not unduly influenced by any single study.

**Conclusion:**

Serum BDNF levels serve as potential biomarkers in patients with depression, but their sensitivity to short-term antidepressant treatment is limited.

## Introduction

Depression is a prevalent psychiatric disorder characterised by persistent mood disturbances, cognitive impairment and neurobiological changes and affects millions of people worldwide [1, 2, 3, 4]. Despite advances in pharmacological and psychotherapeutic interventions, the exact biological mechanisms of depression remain unclear. Brain-derived neurotrophic factor (BDNF) is a secreted growth factor that promotes the regeneration of nerves and the development and plasticity of the nervous system mainly through tyrosine kinase receptor B (TrkB) [5, 6]. In mouse models of social stress, BDNF levels were found to be elevated and BDNF–TrkB signalling leads to depressive-like outcomes [7, 8, 9]. As a neurotrophic critical for neuronal survival and plasticity, BDNF has emerged as a potential biomarker for depression and its treatment outcomes [10, 11]. Increasing evidence suggests that peripheral BDNF levels are decreased in patients with depression and tend to rise substantially after antidepressant treatment [12, 13, 14]. Antidepressants, including selective serotonin reuptake inhibitors (SSRIs) and serotonin-norepinephrine reuptake inhibitors (SNRIs), act partly by upregulating BDNF, promoting the activity of brain regions involved in mood regulation (e.g. hippocampus) and enhancing synaptic remodelling [15, 16, 17].

Although the association between BDNF levels and depression is well documented, the consistency and reliability of BDNF as a biomarker remain controversial. Variations in measurement methods, such as the use of serum versus plasma, patient characteristics and treatment regimens have led to inconsistent results [18, 19]. Furthermore, the temporal dynamics of BDNF during antidepressant treatment remain poorly understood. Some studies reported significant increases in BDNF after 6 weeks of treatment, whereas other studies failed to detect such changes over shorter periods [20, 21].

This study aimed to address the above research gaps by conducting a comprehensive meta-analysis to assess BDNF levels and response to antidepressant treatment in patients with depression. By synthesising the results of different studies, the present study sought to gain a comprehensive understanding of the potential use of BDNF as a diagnostic and therapeutic biomarker.

## Materials and Methods

### Search Strategy

A literature search was conducted in multiple authoritative medical and biomedical literature databases, including PubMed, Embase, Wiley Library, Web of Science, Cochrane library, China National Knowledge Infrastructure, Wanfang Database (Wanfang) and VIP Database. The retrieved documents were limited to Chinese and English texts, and the data update time was as of November 15, 2024. Chinese search terms included the following: ‘BDNF’, ‘brain-derived neurotrophic factor’, ‘depression’ and ‘biomarkers’. The specific search strategy was (‘BDNF’ OR ‘brain-derived neurotrophic factor’) AND (‘depression’ OR ‘depression’) OR (‘biomarker’). The English search terms include: ‘brain derived neurotrophic factor’, ‘BDNF’ and ‘depression’, and the search strategy is (‘brain derived neurotrophic factor’ OR ‘BDNF’) AND (‘major depression’ OR ‘major depressive disorder’ OR ‘MDD’ OR ‘depressive episode’ OR ‘depression’). Although our search primarily used free-text terms for cross-database consistency, database-specific controlled vocabularies (PubMed MeSH, Emtree in Embase) were supplemented where applicable. The detailed search strategy for each database is provided in **Supplementary File 1**.

### Selection Criteria

The inclusion criteria were as follows: (1) for participants, patients with a clinically confirmed diagnosis of depression according to recognised diagnostic criteria (e.g. Diagnostic and Statistical Manual of Mental Disorders, 4th Edition (DSM-IV), Mini-International Neuropsychiatric Interview (MINI) and Edinburgh Postnatal Depression Scale (EPDS)), regardless of gender or the severity or kind of illness; (2) for intervention: studies evaluating changes in serum or plasma BDNF levels in depressed patients before and after antidepressant treatment or comparing BDNF levels between depressed patients and healthy controls (studies reporting plasma BDNF levels were included only for subgroup or comparative analysis due to their limited analytical consistency and high susceptibility to pre-analytical variability); and (3) for treatment data, studies must thoroughly describe the use of antidepressants and treatment time points.

The exclusion criteria were as follows: (1) non-original studies, such as reviews and conference abstracts; (2) non-standard treatments, that is, studies where patients received non-standard depression treatments or interventions unrelated to antidepressant therapy; (3) incomplete data, such as studies with missing or insufficient clinical data, including lack of key variables (e.g. BDNF means/SD, sample size or treatment duration); (4) single-arm studies without baseline control or comparison groups; (5) studies with irrelevant outcome measures, such as those not reporting pre/post BDNF change or comparisons with controls; (6) studies involving patients with severe comorbid conditions (e.g. neurodegenerative diseases, autoimmune disorders) known to affect BDNF; (7) studies using concurrent physical interventions (e.g. electroconvulsive therapy); and (8) Gray literature due to lack of methodological transparency.

### Screening Methods and Data Collection

Two independent researchers initially screened the studies by reviewing abstracts and titles to exclude irrelevant or ineligible papers. Full-text articles were then reviewed against the inclusion criteria to confirm eligibility. Disagreements were resolved through third-party arbitration or consensus discussion. For the final included studies, the two researchers independently collected relevant data, including study design; age; sex; depression severity by Hamilton Depression Rating Scale/Beck Depression Inventory; BDNF sample type, assay method and units; and drug class, duration and response rates. The collected data were cross-verified to ensure their accuracy and consistency. Any inconsistencies were resolved through discussion or consultation with experts. For studies reporting multiple timepoints (e.g. 4-week and 6-week measurements), data were extracted separately and analysed in distinct subgroups to avoid overlap. Each timepoint was treated as an independent dataset in the meta-analysis.

### Quality Assessment

The quality of the 13 included studies—all of which were randomised controlled trials (RCTs)—was independently assessed by two reviewers using the Cochrane Risk of Bias Tool. This tool evaluates the following seven domains: (1) random sequence generation, (2) allocation concealment, (3) blinding of participants and personnel, (4) blinding of outcome assessment, (5) incomplete outcome data, (6) selective reporting and (7) other potential sources of bias. Each domain was rated as ‘low risk’, ‘high risk’ or ‘unclear risk’ of bias. Any disagreements in the assessments were resolved by discussion or consultation with a third reviewer.

### Statistical Analysis

NoteExpress 3.2 software (Aegean Software, Beijing, China) was used for literature management, and Excel 2003 software (Microsoft Corporation, Redmond, WA, USA) was used to collect and extract literature data. Meta-analysis was performed using Revman 5.4.1 software (The Cochrane Collaboration, London, UK). Heterogeneity analysis was performed on the extracted data through Q test (*p* value), and the degree of heterogeneity was evaluated based on the I^2^ value. When *p* > 0.10 or I^2^ ≤ 50%, significant heterogeneity was not present, and the fixed effects model (FEM) was selected for analysis. Otherwise, it indicated the existence of heterogeneity, and the random effects model (REM) was used for analysis. The results of data pooling analysis were described by odds ratio (OR) or mean difference (MD), and their 95% confidence intervals (CI) and forest plots were drawn. Sensitivity analysis was used to test the stability of the meta-analysis results. The significance level of the test was set at α = 0.05 (two-sided).

## Results

### Literature Search Results

**Fig. 1.**
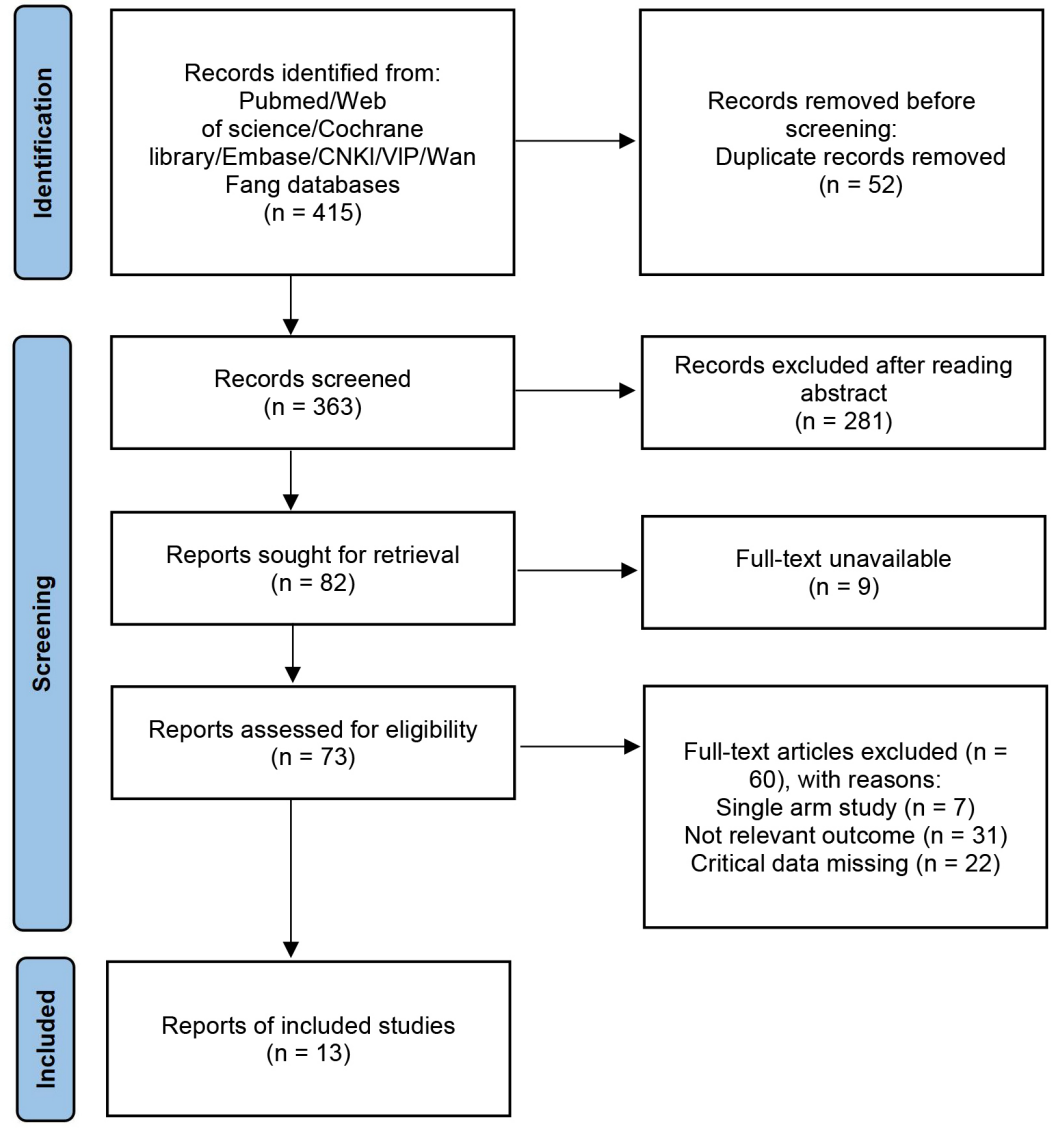
**PRISMA 2020 flow diagram illustrating the study selection process**. CNKI, China National Knowledge Infrastructure.

A total of 415 potentially relevant records regarding serum BDNF levels as biomarkers of treatment response in patients with depression were initially retrieved from Chinese and English databases. After 52 duplicate documents were removed with through literature management software, 363 articles remained. During the title and abstract screening, 281 records were excluded for being clearly irrelevant to the topic, leaving 82 articles for full-text evaluation. During further screening, 9 articles could not be retrieved, and 60 studies were excluded based on predefined criteria including insufficient clinical data, irrelevant outcomes, single-arm study designs and major comorbidities. Finally, 13 studies met the inclusion criteria and were included in the meta-analysis (Fig. 1). The general information about the included studies is listed in Table 1 [21, 22, 23, 24, 25, 26, 27, 28, 29, 30, 31, 32, 33].

**Table 1.** **Criteria characteristics of included studies**.

Study	Year	Country	Sample	Age (year, mean ± SD)	Diagnostic	Antidepressants
Brunoni *et al*. [21]	2014	Brazil	18	41.0 ± 1.0	MINI	SSRI
Karege *et al*. [22]	2005	Geneva	24	31 ± 11 (Control)/36 ± 10 (Patients)	Not mentioned	Not mentioned
Gelle *et al*. [23]	2021	France	85	42	Not mentioned	SSRI, SNRI
Lee *et al*. [24]	2021	Korea	104	32.36 ± 2.98	EPDS	Not mentioned
Ladea and Bran [25]	2013	Romania	20	36.6 ± 8.1	DSM-IV	SSRI
Shimizu *et al*. [26]	2003	Japan	4	46.0 ± 12.2	DSM-IV	SSRI, SNRI
Aydemir *et al*. [27]	2005	Turkey	10	31.8 ± 14.3	DSM-IV	SNRI
Aydemir *et al*. [28]	2006	Turkey	20	35.55 ± 7.58	DSM-IV	SNRI
Hellweg *et al*. [29]	2008	Germany	20	50.5 ± 14.4	DSM-IV	TCA, SSRI
Wolkowitz *et al*. [30]	2011	USA	30	39.1 ± 9.6	DSM-IV	SSRI
Martocchia *et al*. [31]	2014	Switzerland	5	74.0 ± 6.8	DSM-IV	SSRI
Yoshimura *et al*. [32]	2007	Japan	42	46.0 ± 20.5	DSM-IV	SSRI, SNRI
Deuschle *et al*. [33]	2013	Germany	56	52.3 ± 15.9	DSM-IV	SNRI, Mirtazapine

Note: MINI, Mini-International Neuropsychiatric Interview; EPDS, Edinburgh Postnatal Depression Scale; DSM-IV, Diagnostic and Statistical Manual of Mental Disorders, 4th Edition; SSRI, Selective Serotonin Reuptake Inhibitor; SNRI, Serotonin-Norepinephrine Reuptake Inhibitor; TCA, Tricyclic Antidepressant; ‘Not mentioned’ indicates that the original study did not specify this information.

### Quality Evaluation

**Fig. 2.**
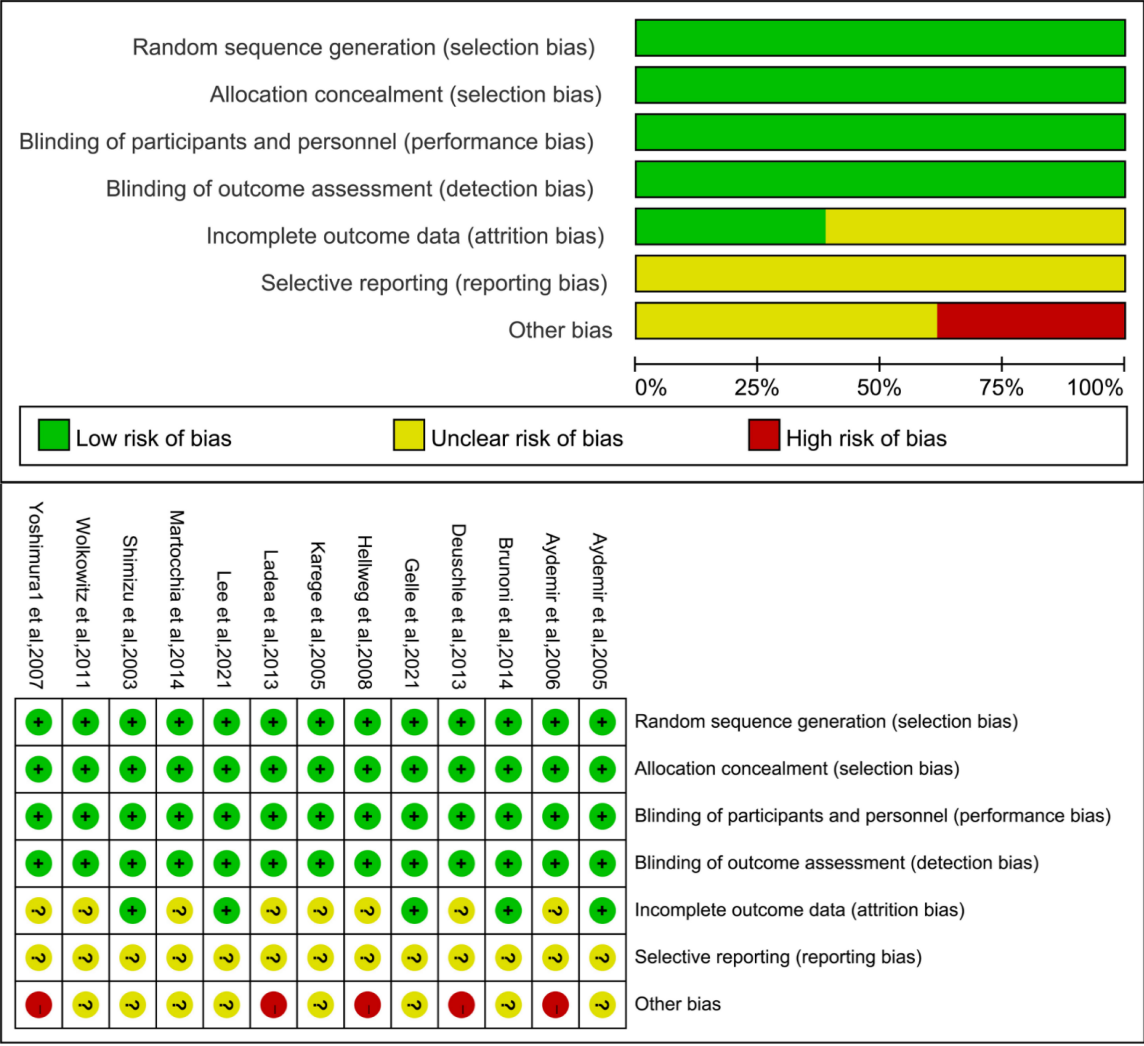
**Cochrane Risk of Bias assessment for the 13 included randomised controlled trials (RCTs)**.

Quality assessment of the included studies (Fig. 2) revealed that all strictly followed the design principles of RCTs. The randomisation methods were clearly described in most works, ensuring the comparability of baseline characteristics between the experimental and control groups. Most of the studies had double-blind designs and specified whether selective reporting was present. However, ambiguity arose regarding whether other biases exist in eight documents, and other bias issues existed in five documents. Overall, the quality of the included works met the requirements of the present study.

### Serum BDNF

**Fig. 3.**
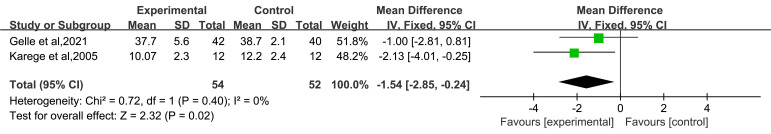
**Forest plot comparing serum BDNF between the two groups of patients**. Note: BDNF, Brain-Derived Neurotrophic Factor. Green squares represent study-specific effect sizes; the black diamond represents the pooled estimate.

Two of the included articles reported serum BDNF levels in the two research groups [22, 23]. Heterogeneity analysis revealed I^2^ = 0% and *p* = 0.40, indicating no statistical heterogeneity among the included studies. Therefore, the FEM was used for analysis. Meta-analysis results showed that the serum BDNF levels of patients with depression were significantly lower compared with those of the control group [MD = –1.54, 95% CI (–2.85 to –0.24), *p* = 0.02]. This result suggests the potential of serum BDNF levels to serve as a candidate biomarker of treatment response in patients with depression (Fig. 3).

### Plasma BDNF

**Fig. 4.**
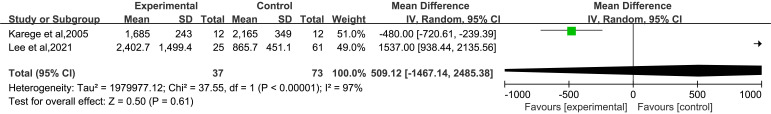
**Forest plot comparing plasma BDNF between two patient groups**. Note: BDNF, Brain-Derived Neurotrophic Factor. Green squares represent study-specific effect sizes; the black diamond represents the pooled estimate.

Two of the included articles reported the plasma BDNF levels of the two groups of research subjects [22, 24] (Fig. 4). Heterogeneity analysis revealed I^2^ = 97% and *p* < 0.00001, indicating heterogeneity among the included studies. Therefore, the REM was used for analysis. Meta results showed that the difference in plasma BDNF levels between the two groups was not statistically significant (*p* > 0.05), indicating that plasma BDNF levels cannot be used as a biomarker of treatment response in patients with depression.

### Total Effect of Serum BDNF Levels After Antidepressant Drug Treatment

**Fig. 5.**
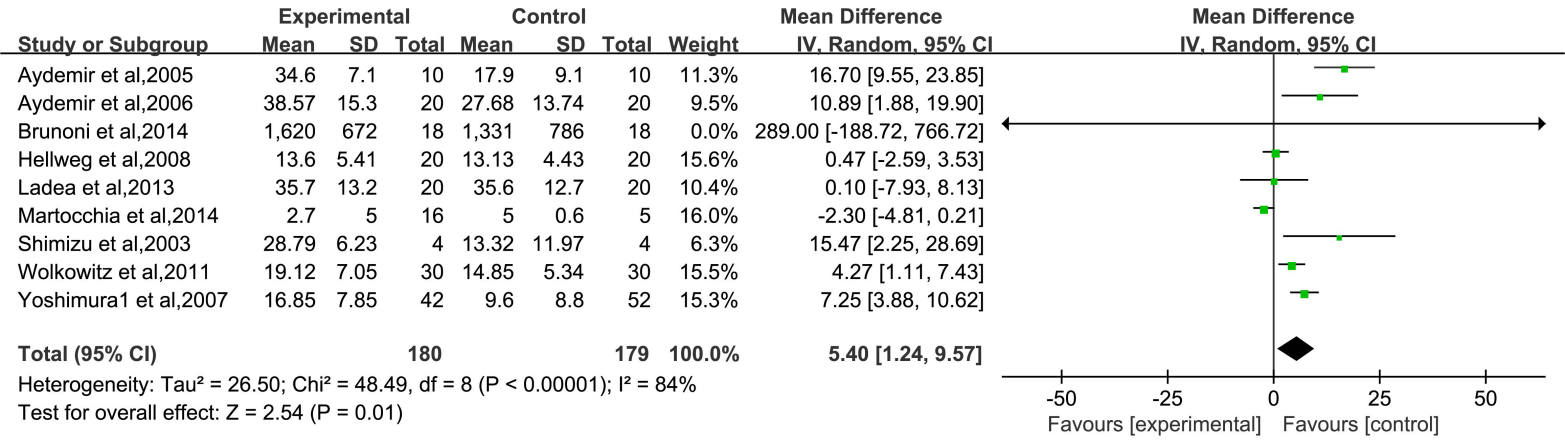
**Forest plot of the total effect of serum BDNF levels after antidepressant drug treatment**. Note: BDNF, Brain-Derived Neurotrophic Factor. Green squares represent study-specific effect sizes; the black diamond represents the pooled estimate.

Fig. 5 shows that nine of the included articles reported BDNF levels before and after treatment (less than 12 weeks) with different types of antidepressants [21, 25, 26, 27, 28, 29, 30, 31, 32]. Heterogeneity analysis revealed I^2^ = 84% and *p* < 0.00001, indicating significant heterogeneity among the studies. Therefore, the REM was used for analysis. Meta-analysis results showed that the serum BDNF levels of patients with depression in the experimental group were significantly increased after antidepressant drug treatment compared with those before treatment [MD = 5.40, 95% CI (1.24–9.57), *p* = 0.01].

### Effects of Antidepressants on Serum BDNF Levels After 4 Weeks of Treatment

**Fig. 6.**
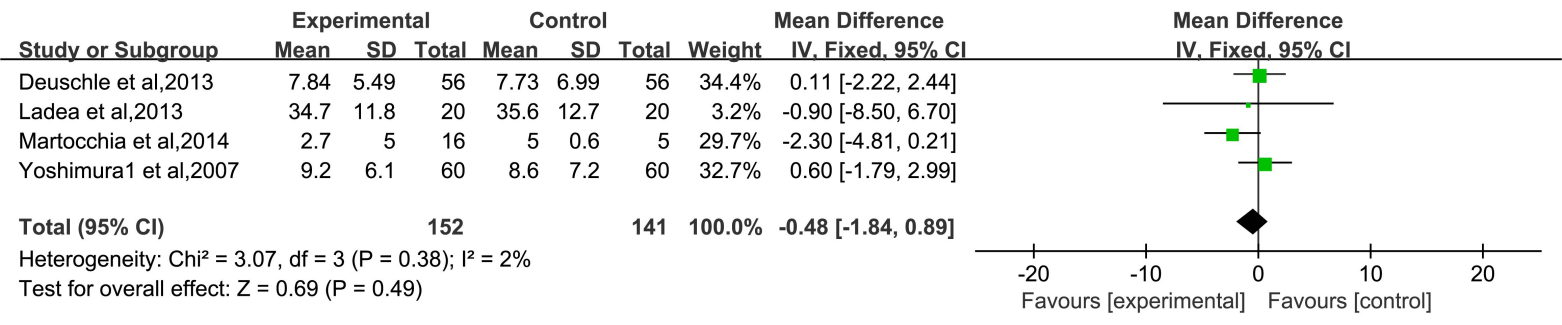
**Forest plot of the effect of antidepressants on serum BDNF concentration after 4 weeks of treatment**. Note: No statistically significant difference was observed (*p* > 0.05). BDNF, Brain-Derived Neurotrophic Factor. Green squares represent study-specific effect sizes; the black diamond represents the pooled estimate.

Four of the included articles reported the effects of 4-week antidepressant treatment on BDNF concentrations in two research groups [25, 31, 32, 33] (Fig. 6). Heterogeneity analysis revealed I^2^ = 2% and *p* = 0.38, indicating the lack of heterogeneity among the included studies. Therefore, the FEM was used for analysis. Meta analysis results showed that the effect of antidepressants on BDNF levels after 4 weeks of treatment was not statistically significant (*p* > 0.05). The lack of significant changes in serum BDNF at 4 weeks may reflect the time needed for downstream neuroplasticity mechanisms to activate.

### Effects of Antidepressants on Serum BDNF Levels After 6 Weeks of Treatment

**Fig. 7.**
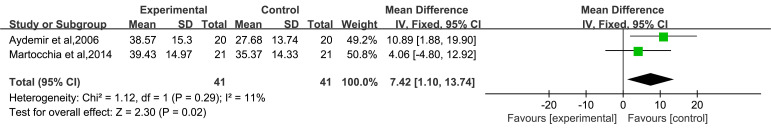
**Forest plot of the effect of antidepressant treatment on serum BDNF levels after 6 weeks**. Note: BDNF, Brain-Derived Neurotrophic Factor. Green squares represent study-specific effect sizes; the black diamond represents the pooled estimate.

As shown in Fig. 7, two of the included documents reported the effect of antidepressants on BDNF levels in two patient groups after 6 weeks of treatment [28, 31]. Heterogeneity analysis revealed I^2^ = 11% and *p* = 0.29, indicating the lack of heterogeneity among the included studies. Therefore, the FEM was used for analysis. Meta analysis results showed that compared with those of the control group before treatment, antidepressant treatment significantly increased serum BDNF levels after 6 weeks [MD = 7.42, 95% CI (1.10–13.74), *p* = 0.02].

### Sensitivity Analysis

**Fig. 8.**
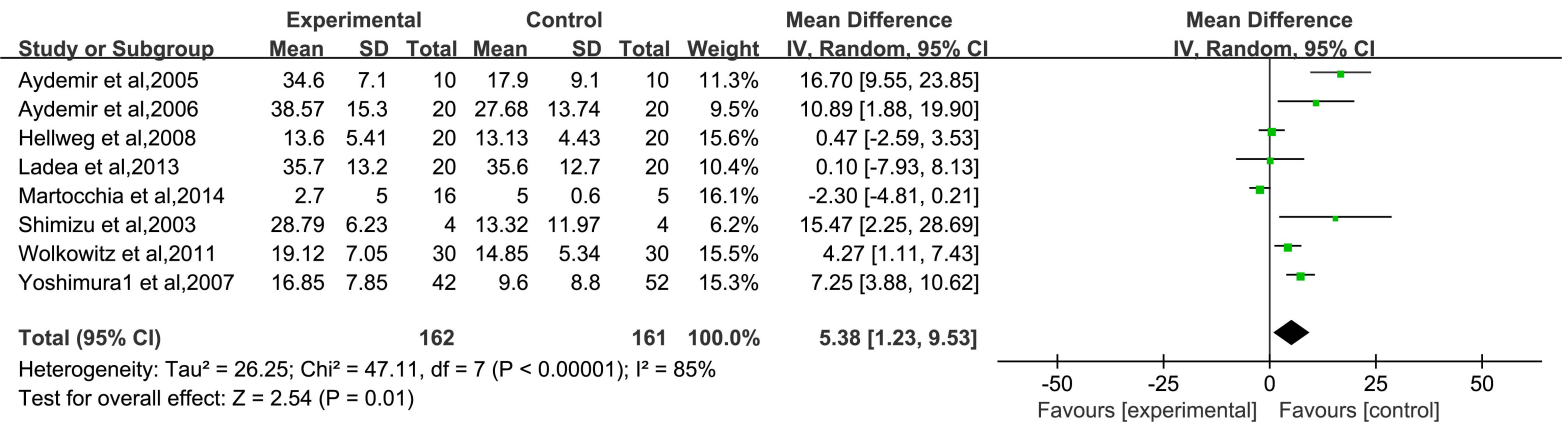
**Forest map for sensitivity analysis**. Note: BDNF, Brain-Derived Neurotrophic Factor. Green squares represent study-specific effect sizes; the black diamond represents the pooled estimate.

Leave-one-out sensitivity analysis was conducted on eight studies that provided complete longitudinal treatment response data to assess the stability and reliability of the meta-analysis results (Fig. 8). Five studies were excluded from this analysis because they either reported only baseline case-control comparisons (n = 2) or lacked complete time-point information (n = 3).

In this analysis, each study was sequentially removed, and the meta-analysis was recalculated to examine the study’s influence on the overall effect sizes. The results showed that exclusion of any single study, including those with small sample sizes (n ≤ 5), did not materially affect the direction or magnitude of the pooled effect estimates. CIs remained relatively stable throughout, indicating that the overall conclusions are not unduly driven by any individual study.

These findings supported the robustness of our model and suggested that the inclusion of small-sample studies did not introduce substantial bias into the meta-analysis results.

## Discussion

This study systematically evaluated how serum and plasma BDNF levels change in patients with depression and how antidepressant treatment affects them through meta-analysis. On the basis of the included studies, the present work summarised the correlation among serum BDNF, plasma BDNF and treatment response in patients with depression and further explored the temporal dynamics and consistency of BDNF level changes after antidepressant drug treatment. Results showed that serum BDNF levels were significantly low in healthy controls and were significantly increased after antidepressant treatment, especially after 6 weeks of treatment.

Firstly, this study showed that serum BDNF levels in patients with depression were significantly lower than those in healthy controls, which is consistent with previous findings. Duman *et al*. [34] pointed out that the decrease in BDNF levels may be closely related to the neurobiological mechanisms of depression. Specifically, BDNF plays a key role in neurogenesis, synaptic plasticity and neuroprotection; a lack of BDNF may lead to neuronal death and dysfunction in specific brain areas, such as the hippocampus [35, 36, 37]. Our analysis results also showed that serum BDNF levels were significantly correlated with treatment response in patients with depression, suggesting that serum BDNF can be used as a potential biomarker to monitor the progress of depression treatment [38]. These findings highlighted the potential of serum BDNF as a state marker of depression and a predictive and monitoring biomarker in clinical practice. For instance, low baseline BDNF levels may help identify patients less likely to respond to conventional antidepressants, guiding early intervention strategies [39]. Furthermore, dynamic changes in BDNF levels during treatment could support biomarker-driven therapy personalisation, aligning with emerging paradigms in precision psychiatry [40].

Despite the significance of the changes in serum BDNF levels, similar differences were not observed for plasma BDNF, which is consistent with some previous studies. This serum–plasma discrepancy may reflect either the biological differences between platelet-derived BDNF and circulating BDNF or the methodological factor of plasma having a lower concentration and greater pre-analytical variability. Carvalho *et al*. [41] reported that plasma BDNF levels cannot be used to reliably differentiate depressed patients from healthy controls, suggesting that plasma BDNF may not be used as an independent biomarker of depression. Relevant studies also pointed out that the measurement of plasma BDNF levels may be affected by a variety of factors (such as sampling methods and molecular complexity in plasma) and therefore may not stably reflect changes in the central nervous system [42, 43, 44]. Our present work showed that the reliability of BDNF as a biomarker may be affected by the timing and method of treatment. Different types of antidepressants may affect BDNF expression through different mechanisms, and these effects may not be significant enough in the short term [45].

After antidepressant drug treatment, the changes in BDNF levels reflect the promoting effect of drug intervention on neuroplasticity and neurogenesis. Although SSRIs and SNRIs both enhance BDNF, their distinct neurotransmitter targets (serotonin vs. norepinephrine) may lead to their differential temporal patterns of BDNF regulation, thus warranting further investigation. Our meta-analysis showed that BDNF levels significantly increased after 6 weeks of antidepressant treatment, and this change was consistent with the mechanism of action of antidepressants. Drugs such as SSRIs and SNRIs usually promote neurogenesis and synaptic remodelling in brain regions related to emotion regulation (such as the hippocampus and prefrontal cortex) by upregulating BDNF [46]. However, the results after 4 weeks of treatment did not show significant changes, suggesting that BDNF upregulation may take a long time to manifest therapeutic effects. This finding also provided support for the time dependence of antidepressant drug efficacy. The delayed BDNF elevation (significant at 6 weeks but not at 4 weeks) mirrors the typical latency of antidepressant clinical effects, suggesting that BDNF may be more closely associated with sustained therapeutic responses than initial drug actions.

Compared with prior meta-analyses [18, 19], our findings confirmed the post-treatment increase in serum BDNF and provided more temporally precise evidence by demonstrating that significant changes emerged at 6 weeks, not 4 weeks. This distinction supports the view that BDNF elevation reflects sustained (rather than early) treatment effects, aligning with the known delay in clinical antidepressant response. This discrepancy may reflect our strict inclusion criteria and the use of subgroup analysis based on treatment duration, allowing us to distinguish short-term (4-week) from intermediate-term (6-week) responses. Additionally, we confirmed the superior reliability of serum BDNF as a treatment-responsive biomarker by separately analysing serum and plasma BDNF—rather than pooling them [40]. Our inclusion of the most recent studies further enhances the timeliness and relevance of our findings. These methodological refinements allow our study to supplement and strengthen existing evidence with great clarity and precision.

Despite the inclusion of a relatively large number of studies, this meta-analysis has several limitations that should be acknowledged. One major concern is the presence of substantial heterogeneity across studies, possibly driven by methodological differences in BDNF measurement protocols (serum versus plasma, assay types), variation in patient characteristics (such as age, sex and depression subtype) and use of different antidepressant classes (e.g. SSRIs, SNRIs and tricyclics). Although subgroup analyses or meta-regression could have clarified these sources, the limited number of studies and incomplete reporting of key covariates precluded such approaches. Additionally, some studies did not provide enough information or adopt a non-double-blind design, which may affect the reliability of the research results. Moreover, some subgroup analyses (e.g. plasma BDNF, 6-week treatment effects) included only two studies, limiting the precision of these specific estimates. Publication bias could not be formally assessed, as most comparisons included fewer than 10 studies—below the minimum threshold for reliable funnel plot interpretation or statistical tests such as Egger’s regression. Hence, the risk of publication bias remains unclear. Future research should include large, high-quality trials to enable robust bias detection and quantitative synthesis. Standardisation of BDNF assessment protocols and stratification by treatment duration and drug class are also recommended to improve cross-study comparability and clinical applicability.

## Conclusion

This study conducted a systematic review and meta-analysis to evaluate BDNF expression levels in patients with depression and its potential predictive value for antidepressant treatment response. Results demonstrated that serum BDNF levels were significantly lower in patients with depression that in healthy controls and were increased significantly following 6 weeks of antidepressant treatment, suggesting the potential utility of serum BDNF as a biomarker of treatment response. By contrast, plasma BDNF did not show statistically significant changes, indicating its limited sensitivity and specificity. Future studies should aim to standardise BDNF detection methods and sample processing protocols; conduct large-scale, multicentre, prospective trials; and further explore the temporal dynamics of BDNF changes under varying treatment durations to facilitate its clinical translation in the precision treatment of depression.

## Availability of Data and Materials

The data used to support the findings of this study are available from the corresponding author upon request.

## Supplementary Material

Supplementary material associated with this article can be found, in the online version, at https://doi.org/10.62641/aep.v53i4.1967.
