# Supplementary material for: Serum Brain-derived Neurotrophic Factor Levels as a Biomarker of Treatment Response in Patients With Depression: Systematic Review and Meta-analysis
Source: Actas Esp Psiquiatr. 2025 Aug 5;53(4):857–67. doi: 10.62641/aep.v53i4.1967 (PMC12353246; doi:10.62641/aep.v53i4.1967)
Supplement: Supplementary file 1 [file ActEsp-53-4-857-867-s1.zip › Supplementary File 1.docx]

**Supplementary File 1. Detailed Literature Search Strategy**

1. PubMed
   · Search Date: Up to November 15, 2024
   · Language: English
   · Search Strategy:
   *("brain derived neurotrophic factor"[Title/Abstract] OR "BDNF"[Title/Abstract])
   AND*

*("major depression"[Title/Abstract] OR "major depressive disorder"[Title/Abstract] OR "MDD"[Title/Abstract] OR "depressive episode"[Title/Abstract] OR "depression"[MeSH Terms])*

1. Database: Embase
   · Search Date: Up to November 15, 2024
   · Language: English
   · Search Strategy:
   *('brain derived neurotrophic factor'/exp OR 'BDNF') AND ('depression'/exp OR 'major depressive disorder')*
2. Web of Science Core Collection

· Search Date: Up to November 15, 2024

· Language: English

· Search Strategy (Topic search):

*TS=("brain derived neurotrophic factor" OR BDNF)*

*AND*

*TS=("major depression" OR "major depressive disorder" OR MDD OR "depressive episode" OR depression)*

1. Wiley Online Library

· Search Date: Up to November 15, 2024

· Language: English

· Search Strategy:

*("brain derived neurotrophic factor" OR BDNF)*

*AND*

*("major depressive disorder" OR depression OR MDD)*

1. Cochrane Library (CENTRAL)

· Search Date: Up to November 15, 2024

· Language: English

· Search Strategy:

*("brain derived neurotrophic factor" OR BDNF):ti,ab,kw*

*AND*

*("depression" OR "depressive disorder" OR MDD):ti,ab,kw*

1. CNKI
   · Search Date: Up to November 15, 2024
   · Language: Chinese
   · Search Strategy:

*("脑源性神经营养因子" OR "BDNF") AND ("抑郁症" OR "抑郁障碍")*

1. Wanfang Database

· Search Date: Up to November 15, 2024
· Language: Chinese
· Search Strategy:

*("BDNF" OR "脑源性神经营养因子") AND ("抑郁症" OR "情感障碍")*

1. VIP Database

· Search Date: Up to November 15, 2024
· Language: Chinese
· Search Strategy:

*("脑源性神经营养因子" OR "BDNF") AND ("抑郁症" OR "重性抑郁障碍")*

Note: In all searches, filters were applied to limit results to human studies (where available), and duplicates were removed using literature management software. No publication type filters were applied during the initial search to ensure sensitivity.
